# Preclinical study of diagnostic performances of contrast-enhanced spectral mammography versus MRI for breast diseases in China

**DOI:** 10.1186/s40064-016-2385-0

**Published:** 2016-06-17

**Authors:** Qingguo Wang, Kangan Li, Lihui Wang, Jianbing Zhang, Zhiguo Zhou, Yan Feng

**Affiliations:** Department of Radiology, Shanghai First People’s Hospital, No 100, Haining Road, Hongkou District, Shanghai, China; Department of Pathology, Shanghai First People’s Hospital, Shanghai, China

**Keywords:** Contrast-enhanced spectral mammography, Magnetic resonance imaging, Breast cancer, Pathology

## Abstract

**Purpose:**

To evaluate diagnostic performances of CESM for breast diseases with comparison to breast MRI in China.

**Materials and methods:**

Sixty-eight patients with 77 breast lesions underwent MR and CESM. Two radiologists interpreted either MRI or CESM images, separately and independently. BI-RADS 1–3 and BI-RADS 4–5 were classified into the suspicious benign and suspicious malignant groups. Diagnostic accuracy parameters were calculated. Receiver operating characteristic (ROC) curves were constructed for the two modalities. The agreement and correlation between maximum lesion diameter based on CESM and MRI, or CESM and pathology were analyzed.

**Results:**

Diagnostic accuracy parameters for CESM were sensitivity 95.8 %, specificity 65.5 %, PPV 82.1 %, NPV 90.5 % and accuracy 84.4 %. The diagnostic accuracy parameters for breast MRI were sensitivity 93.8 %, specificity 82.8 %, PPV 88.2 %, NPV 92.3 %and accuracy 89.6 %. Area under the curve (AUC) of ROC was 0.96 for breast MRI and 0.88 for CESM. The Bland–Altman plots showed a mean difference of 0.7 mm with 95 % limits of agreement of 11.4 mm in tumor diameter measured using CESM and breast MRI. The differences of size measurement between CESM and breast MRI were significant, whereas no difference was observed between CESM and pathology as well as between breast MRI and pathology. The better correlation with pathological results was found in CESM than breast MRI.

**Conclusion:**

Our study demonstrates that CESM possesses better diagnostic performances than breast MRI in terms of diagnostic sensitivity and lesion size assessment. And CESM is a good alternative method of screening breast cancer in high-risk people.

## Background

Early detection and diagnosis are essential for the prognosis and treatment of breast cancer. The most commonly used methods include digital mammography (DM), ultrasound (US) and magnetic resonance imaging (MRI).

DM has a low diagnostic sensitivity and specificity in women with dense breasts due to its masking effects (Pisano et al. 2005). The US is usually considered as a supplemental screening method for women with dense breasts to increase the detection of breast cancer. However, hand-held US screening by the radiologist has a high false-positive rate, which may not be a preferable method for breast surgeons (Berg et al. 2008).

At present, MRI is considered the best imaging investigation for the detection and diagnosis of breast cancer (Schell et al. 2009; Pickles et al. 2016; O’Flynn et al. 2016; Iacconi et al. 2015). Breast MRI is cost-effective in screening high-risk women. However, due to high cost and time consuming, preoperative breast MRI can’t be widely available. There may be a high number of false-positive findings in breast MRI which may cause spiritual anxiety and unnecessary treatment.

Contrast enhanced spectral mammography (CESM) is a novel breast imaging technique that has been investigated to display contrast up-take in breast lesions (Diekmann et al. 2005; Diekmann et al. 2011). CESM improves the sensitivity and specificity for breast cancer detection, as it provides higher foci to breast gland contrast and better lesion delineation than DM (Dromain et al. 2011). Preliminary results suggest that, similar to breast MRI, CESM had a good practice for evaluating lesion extent, lesion size and detecting more multifocal lesions. However, so far there are only a few studies with regard to CESM examinations with comparison to breast MRI.

The purpose of our study was to compare bilateral CESM and breast MRI with regard to the diagnosis and size estimation of histologically proven breast diseases using postoperative histology findings as the gold standard. CESM will be considered as a competitive modality if the difference of diagnosis performance is <10 % compared to breast MRI.

## Patients

Our institutional review board approved this prospective study. Written informed consent form was obtained from each patient. The participants should be females presenting for breast diagnostic imaging, either with clinical or previous suspicious imaging findings.

### Inclusion criteria

The participant should be a female over the age of 18 years without the history of mammary gland excision. The participant shouldn’t be pregnant confirmed by negative urine pregnancy test. The participant presents with suspicious breast clinical symptoms and/or previous breast findings classified BI-RADS ≥ 3 on mammography and/or ultrasound. The participant is willing to have all imaging procedures completed (MRI, CESM) within a 3-week interval.

### Exclusion criteria

The breasts of the participant are too large to be adequately demonstrated. The contraindications for contrast agents are applied in this study. The subject is undergoing radiotherapy or chemotherapy. The participant has acute medical condition requiring urgent care. Any conditions judged by the Investigator would interfere with the evaluation of the results or cause a potential hazard to the participant’s health.

## CESM technique and protocol

The CESM equipment was a commercial model provided by GE Healthcare (Senographe DS and Senographe Essential mammography systems, Buc, Cedex, France). An intravenous catheter was inserted in the forearm prior to the examination.

**Fig. 1:**
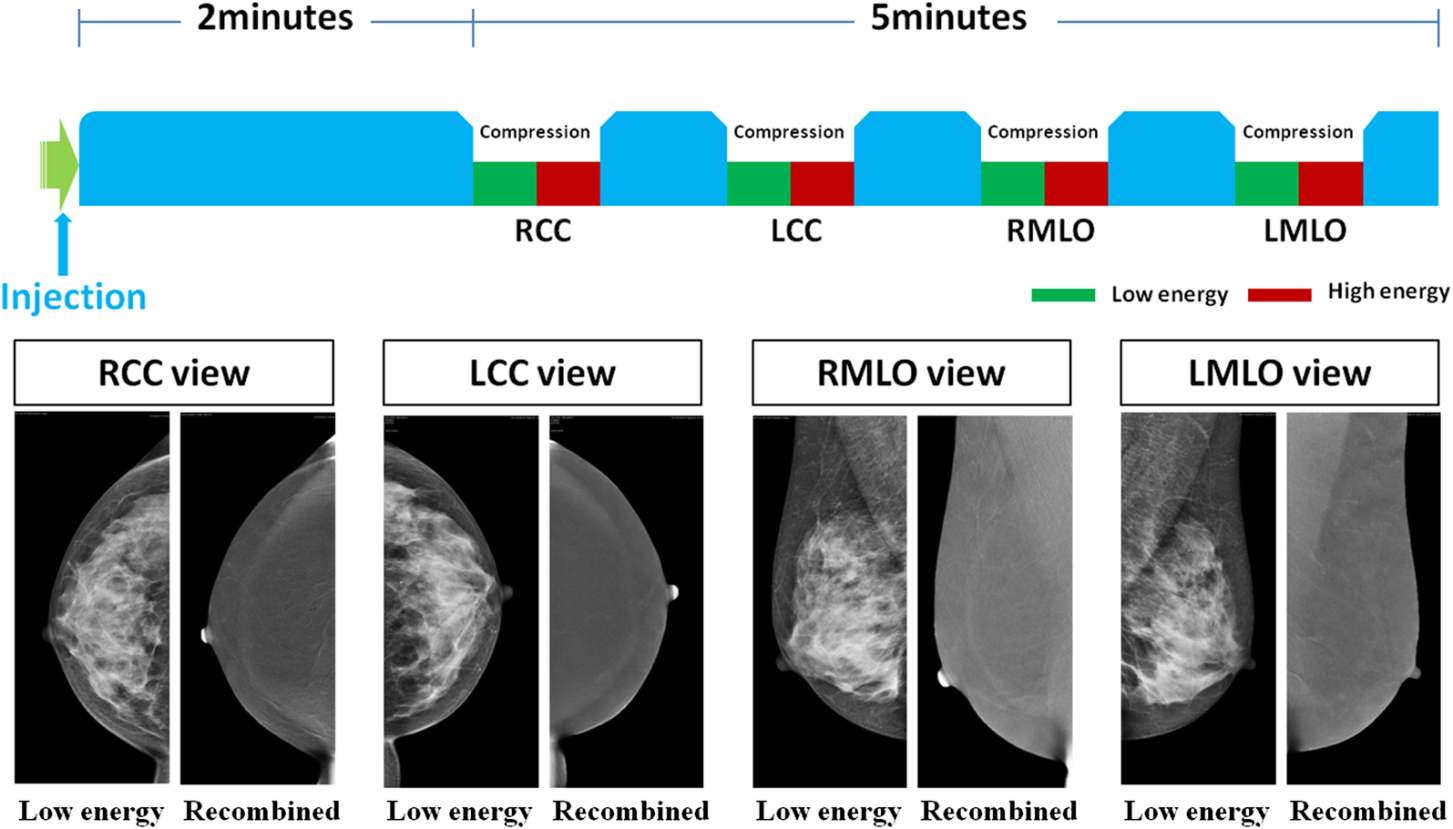
Schematic overview of procedures in CESM. The low-energy and recombined images on CC and MLO in a 45-year-old volunteer are shown according to the order of projection views illustrated in the *top of the figure*. *CC* craniocaudal, *MLO* mediolateral oblique

An 18-gauge catheter was placed into an antecubital vein prior to the examination. A nonionic contrast material (Omnipaque, 350 mgI/mL; GE Healthcare, Dublin, Ireland) at a 1.5 mL/kg, to a maximum of 90 mL was injected at a flow rate of 3.0 mL/s. Consecutive mammogram was performed with craniocaudal (CC) and mediolateral oblique (MLO) views of the bilateral breasts at 2 min after completion of contrast agent injection. All four views completed within 5 min. A pair of low-energy (LE) and high-energy (HE) images is acquired within 1 s under normal mammographic compression in each projection (Fig. 1).

## MR examination

All MR examinations were performed on a 1.5-T MR system (Signa HDx, GE Medical System, Milwaukee, WI, USA) in prone position using a four-channel breast coil.

Diffusion-weighted imaging (b = 800 sec/mm^2^) and dynamic T1w Vibrant 3D gradient echo sequences (repetition time 5.2 ms, echo time 2.1 ms, flip angle 15°, field of view 320–360 mm, 512 × 512 matrix, slice thickness 2.0 mm, no intersection gap). After the unenhanced fat-saturated T1WI, five contrast enhanced image sets with a gap of 1.7-s were acquired with a 15-s delay after starting the contrast injection, resulting in an individual duration for one acquisition of 46–75. Contrast agents (Magnevist^®^, Bayer-Healthcare, Germany) with a dose of 0.1 mmol/kg body weight were injected using an automated syringe at a rate of 2 ml/s as a single intravenous bolus followed by a 20 ml saline flush.

## Image reading method

**Fig. 2:**
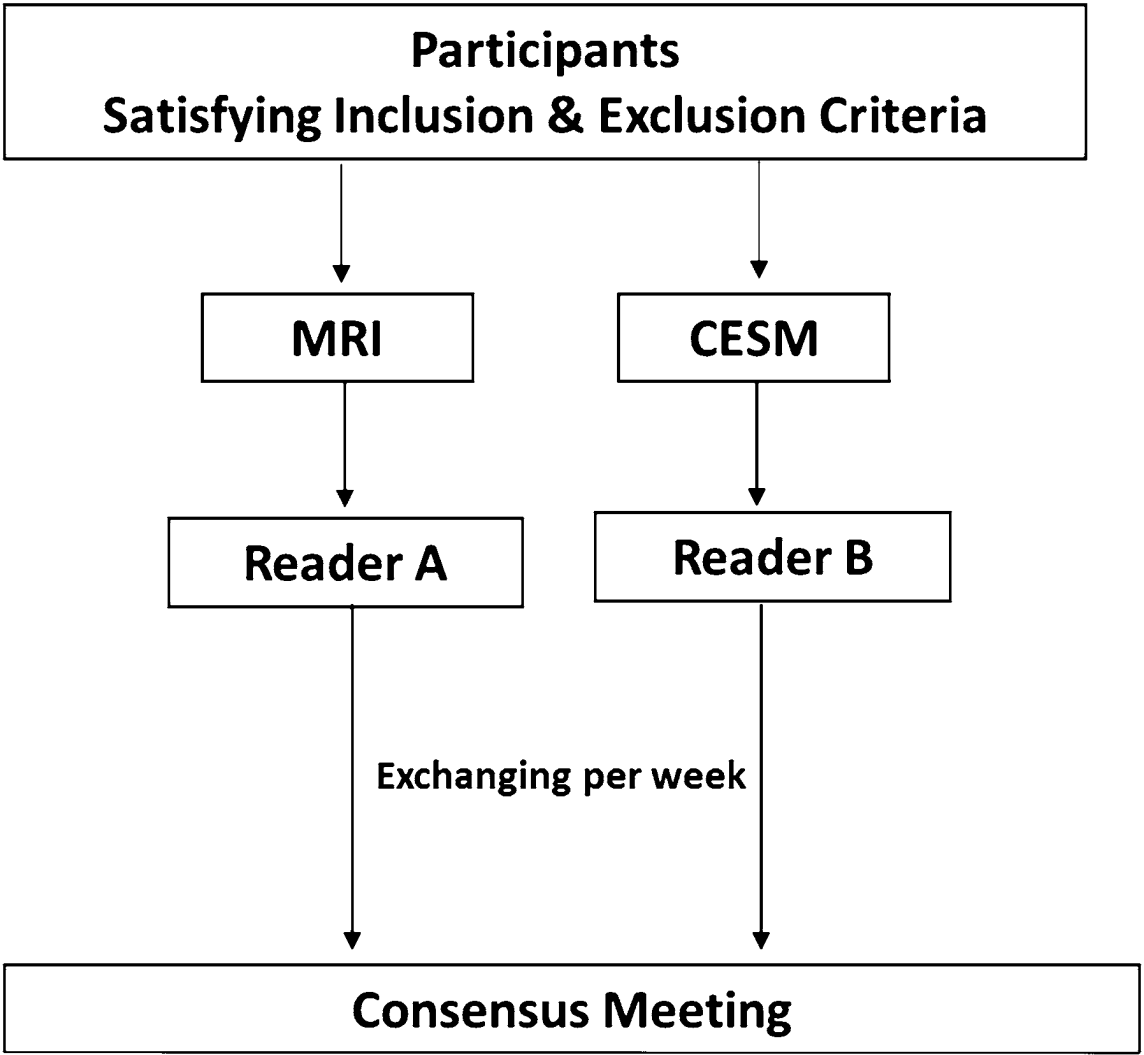
Imaging reading procedures

Our study was a blinded on truth and non-randomized research. The MRI images were anonymized by a study coordinator prior to study readings. The CESM images were anonymized at the time of acquisition with the subjects’ unique study number. Two radiologists were asked to read images. They were blinded to the patients’ clinical data and had no experience with CESM. They interpreted either MRI or CESM images, separately and independently (Fig. 2). MG or ultrasound of the case was not available to the readers. CESM interpretation included both low energy and subtracted (iodine) images. However, they had experience with breast sonography, digital mammography (DM) and breast MRI. The radiologists recorded separately for MRI and CESM the most suspicious finding for each breast of the subject. The two readers involved in the study exchanged their work weekly. Patient management decisions may involve a consensus meeting: if the CESM reading shows additional information versus MRI, the Study Coordinator will organize a consensus meeting between the radiologist in charge of the patient care and the CESM readers. The consensus meeting was organized at the latest 2 weeks after imaging. A consensus decision will be taken on how to use the additional information from CESM on patient management. As such, the radiologist in charge of the patient care may go to existing MRI or MG images or possibly MG special views or suggest a new ultrasound examination to refine the diagnosis, as per usual clinical practice. CESM images were reviewed using IDI^®^ or SenoAdvantage^®^ workstation with CESM review softwares.

## Truth establishment

Truth was derived from tissue sampling and histopathology reports for cases with positive assessments on MRI and/or CESM. Tissue sampling should be completed in maximum 4 weeks after the last study imaging procedure. Cases with negative assessments (BI-RADS ≤ 2) on both MRI and CESM were considered as true negatives and needn’t require histopathology.

## Statistical analysis

For statistical analysis, BI-RADS 1–3 and BI-RADS 4–5 were classified into the suspicious benign and suspicious malignant groups, respectively.

The rates of true positive, true negative, false positive and false negative for malignancy were counted. The diagnostic sensitivity, specificity, positive predictive value (PPV), negative predictive value (NPV) and accuracy were calculated.

The differences were tested for statistical significance using McNemar’s test for paired proportions. Receiver operating characteristic (ROC) curves were constructed for the two imaging modalities and the areas under the curve (AUC) with corresponding 95 % CI were calculated.

Agreement and correlation analysis between maximum lesion diameter based on CESM and MRI, or CESM and surgical specimen was demonstrated by using Bland–Altman plots and Spearman analysis. The difference of maximum lesion diameter between two groups was compared using paired *t* test (SPSS, version 17, IBM, Armonk, NY, USA). P ≤ 0.05 were considered statistically significant.

## Results

A total of 103 patients were initially enrolled in this study. Three patients refused contrast agent administration for CESM. Fifteen patients refused surgical therapy. Sixteen patients had no masses found both on CESM and MRI.

Sixty eight patients with 77 pathologically proven lesions were eventually enrolled in this study. Two patients each had three lesions, two had two lesions and one had four lesions. Thirty six patients were postmenopausal and 32 premenopausal. The patients had a mean age of 52.9 years ranging from 31 to 82 (SD = 10.7). CESM and MRI were available for all patients.

**Fig. 3:**
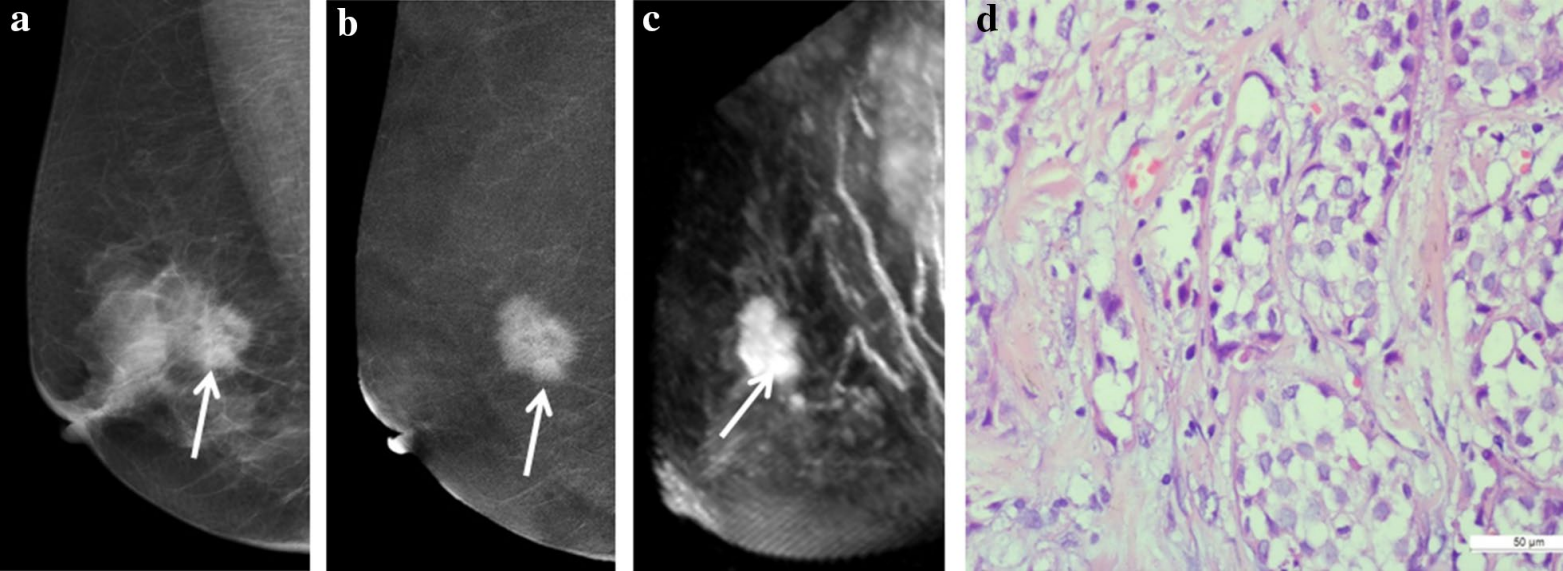
A 62-year-old patient with a 3.5-cm IDC (grade 2) in the right breast. **a** The low-energy image shows a prominent mass on CC. **b** The recombined CESM images show an enhanced mass (*white arrow*) with lobulation and spicule sign on the MLO and CC. **c** The MRI MIP image of subtraction 4 min after contrast injection show obvious enhancement of mass (*white arrow*) with similar appearances to CESM. **d** Microphotograph indicates the diagnosis of IDC lacking myoepithelium (×400, Haematoxylin and eosin)

**Fig. 4:**
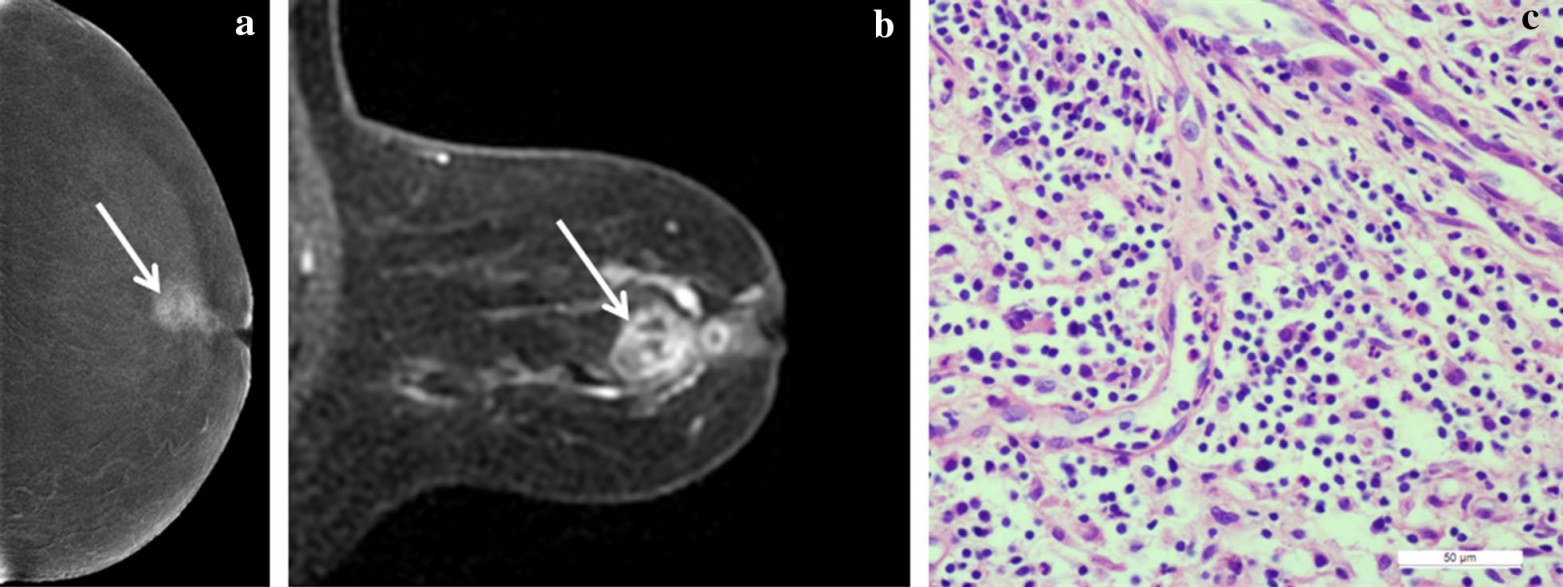
A 64-year-old patient with a 2.5-cm lesion in the left breast. **a** A recombined image on CC shows an enhanced mass (*white arrows*) with several small cystic non-enhanced areas. **b** A MRI MIP image of subtraction shows inhomogeneous enhancement of mass (*white arrows*) similar to recombined CESM images. **c** Microphotograph indicates the diagnosis of inflammatory pseudotumor (×400, Haematoxylin and eosin)

**Fig. 5:**
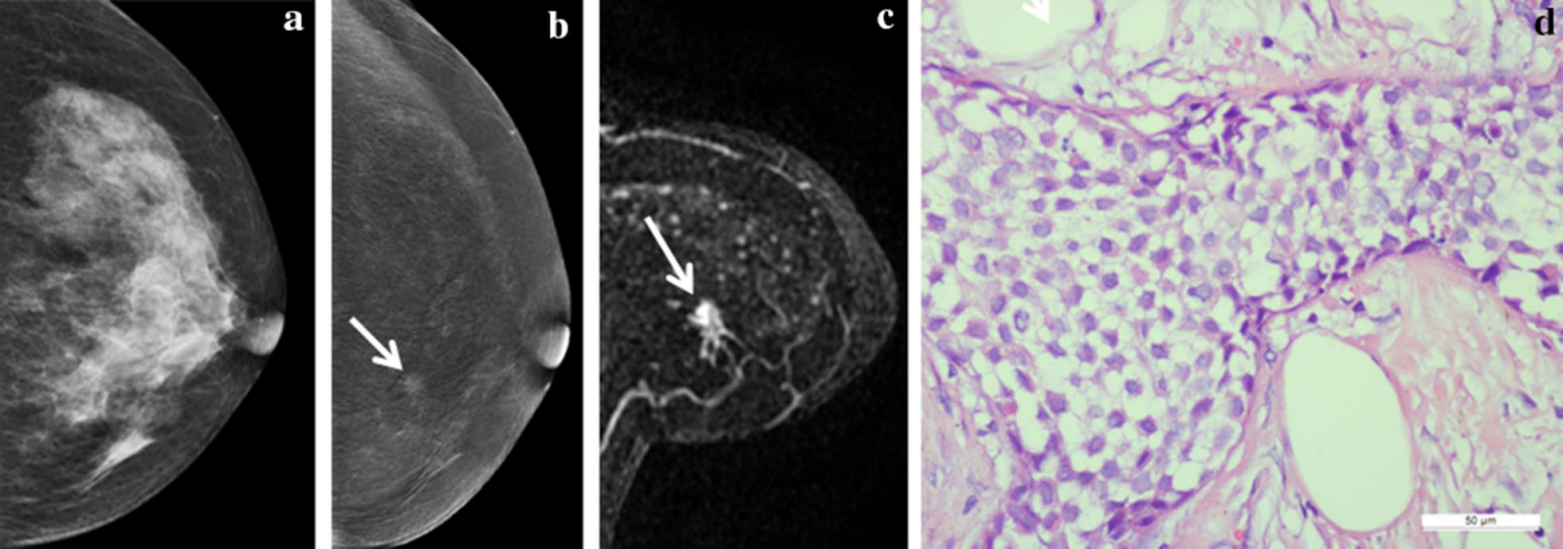
A 47-year-old patient with a 0.4-cm lesion in the left breast. **a** The low-energy image can’t show the lesion on CC. **b** The recombined image shows a small enhanced lesion (*white arrow*) on the upper inner quadrant on the CC. **c** The MRI MIP images of subtraction 4 min after contrast injection show obvious enhancement of the small mass (*white arrow*). **d** Microphotograph indicates the diagnosis of ductal carcinoma in situ. (×400, Haematoxylin and eosin)

Of 77 lesions, 48 lesions were pathologically diagnosed as cancer and 29 as benign disease. The most common pathological type of breast cancer is invasive ductal carcinoma (IDC) (Fig. 3) accounting for 46.8 % (36/77) of all lesions. A detailed overview of pathological diagnosis is presented in Table 1. Diagnostic accuracy parameters for CESM were sensitivity 95.8 % (46/48), specificity 65.5 % (19/29), PPV 82.1 % (46/56), NPV 90.5 % (19/21) and accuracy 84.4 % (65/77). The diagnostic accuracy parameters for MRI were sensitivity 93.8 % (45/48), specificity 82.8 % (24/29), PPV 88.2 % (42/51), NPV 92.3 % (24/26) and accuracy 89.6 % (69/77). CESM detected three cancers that were misdiagnosed as benign lesions by MRI including one intra-cystic papillary carcinoma and two DCIS (Fig. 4). Two inflammatory granulomas (Fig. 5) were diagnosed as breast cancer by both CESM and MRI. Three fibroadenomas were not shown on recombined images whereas clearly shown on low-energy images by using CESM.

**Table 1:** A detailed overview of pathological diagnosis

Malignant lesions (%)		Benign lesions (%)	
Invasive ductal carcinoma (n = 36)	46.8	Fibroadenoma (n = 16)	20.8
Ductal carcinoma in situ (n = 5)	6.5	Adenosis (n = 6)	7.8
Intracystic papillary carcinoma (n = 2)	2.6	Granulomatous inflammation (n = 3)	3.9
Invasive cribriform carcinoma (n = 2)	2.6	Intraductal papilloma (n = 2)	2.6
Mucinous carcinoma (n = 2)	2.6	Atypical lobular neoplasia (n = 1)	1.3
Invasive micropapillary carcinoma (n = 1)	1.3	Duct ectasia with cyst (n = 1)	1.3

Data are percentage (no./total)

## ROC analysis

**Fig. 6:**
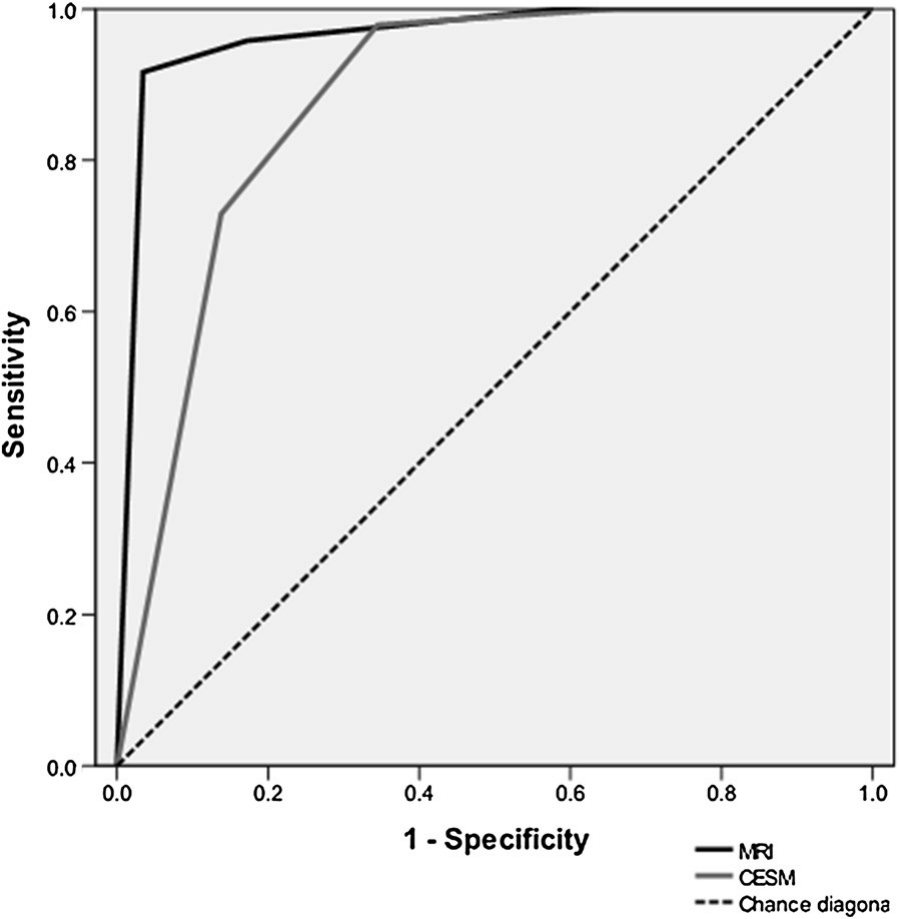
Receiver operating characteristic (ROC) curve for CESM (*grey line*) and MRI (*black line*). The area under curve (AUC) of ROC in breast MRI is larger than that in CESM (z = 2.18, *P* = 0.03)

Area under the curve (AUC) of ROC was 0.96 for MRI and 0.88 for CESM, with a *P* value for the difference of 0.03 (Fig. 6).

## Lesion size assessment

**Fig. 7:**
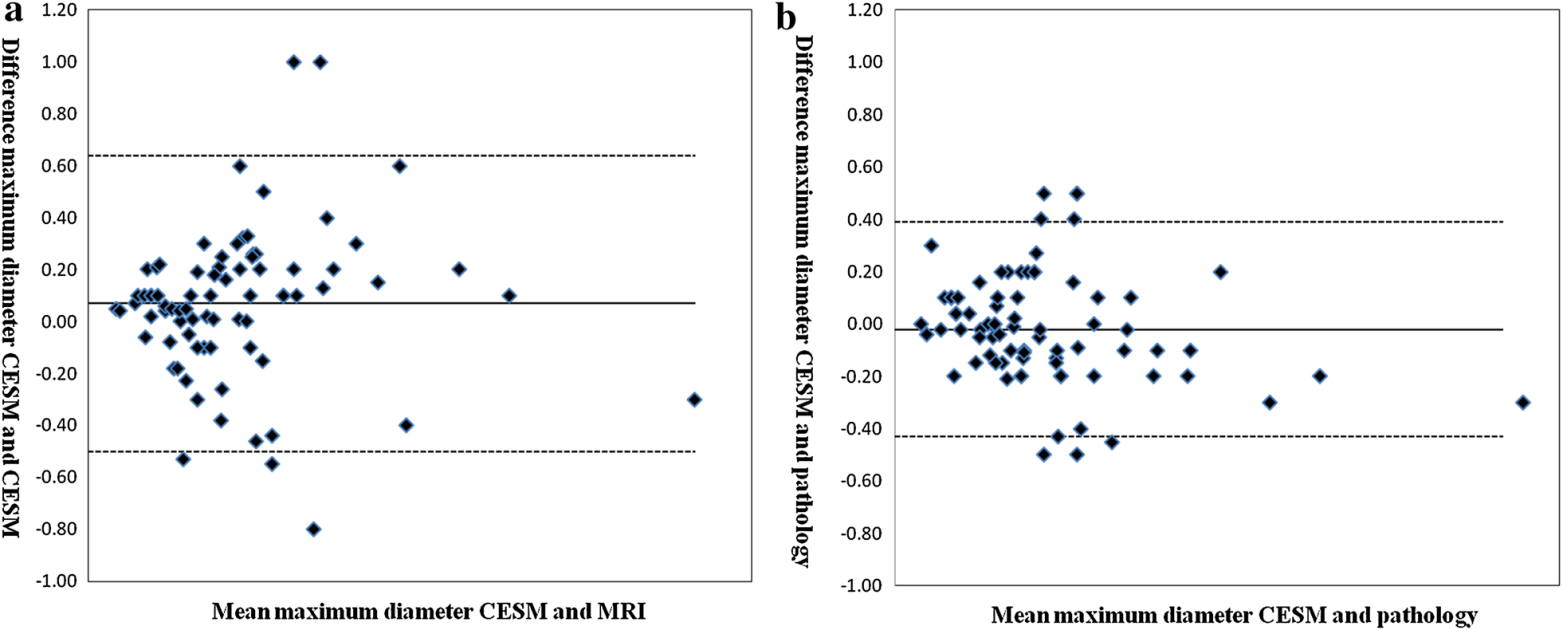
Bland–Altman plots of agreements between a lesion diameter measured using different methods. **a** The *plot* shows the agreement between CESM and MRI. **b** The *plot* shows the agreement between CESM and pathology

The Bland–Altman plots showed a mean difference of 0.7 mm with 95 % limits of agreement of 11.4 mm in tumor diameter measured using CESM and breast MRI (Fig. 6). A mean difference of 0.2 mm was observed between tumor diameters assessed using CESM and histopathology, but with smaller 95 % limits of agreement of 8.2 mm (Fig. 7).

The differences of size measurement between CESM and MRI were significant (*P* < 0.01), whereas no difference was observed between CESM and pathology as well as between MRI and pathology (*P* *>* 0.05). The better correlation with pathology was found for CESM than MRI (*ρ* = 0.975 vs. 0.952).

## Discussion

CESM is a novel breast imaging technique based on dual energy acquisition (Daniaux et al. 2015). A pair of low- and high-energy images is acquired. And the two images are recombined into one image which looks like a digital subtraction image. CESM is an advanced application of conventional mammography. Prionas et al. (2010) confirmed that the iodinated contrast medium showed enhancement of a hypervascular lesion with higher cancer detection than conventional mammography. Cheung et al. (2014) analyzed 110 lesions in 89 patients and showed that CESM provided additional information with consistent improvement of the cancer diagnosis in dense breasts compared to conventional mammography alone. MRI is considered to be the best modality for breast disease diagnosis. When CESM is proposed as a new alternative method for screening and diagnosing breast cancer, the role of breast MRI is challenged. However, the superiority of CESM to MRI is still under debate. Our study demonstrated that the diagnosis accuracy of CESM (84.4 %, 65/77) was inferior to MRI (89.6 %, 69/77) and AUC was 0.96 for MRI and 0.88 for CESM (*P* *<* 0.001). Nevertheless, the sensitivity of CESM (95.8 %, 46/48) appeared superior to that of MRI (93.8 %, 45/48). The better correlation with pathology was found for CESM than MRI in lesion size assessment.

Currently, breast cancer screening is more popular and more available than past in China. The breast tumor can be detected in its very early stage and the lesion size is smaller than before. In our study, the maximum diameter of the smallest tumor was 0.4 cm. Some small lesions were misdiagnosed by using CESM due to lack of characteristics of radiological diagnosis. Therefore, the negative prediction value (NPV) and the AUC was smaller in CESM than breast MRI.

In our study, the specificity of CESM (65.5 %, 19/29) was inferior to that of MRI (82.8 %, 24/29). But Jochelson (2012) showed that the sensitivity of CESM was higher than that of MRI, the specificity had opposite performance. Our outcome may be due to the combined application of DWI and dynamic contrast enhancement in our study, which improved the diagnostic specificity for breast cancer. The lack of experiences in CESM diagnosis for breast disease may be another reason.

The low-energy image of CESM is similar to conventional mammography. We can detect calcifications and the lesions not shown on CESM due to lack of blood supply. In our study, three fibroadenomas were not found on recombined images whereas clearly shown on low-energy images due to poor blood supply. According to our experiences, low-energy images of CESM could provide important complementary information when we are entangled with final diagnosis by using recombined images.

For other diagnostic performances, CESM was better in size assessment than MRI. For CESM versus histopathology, the Pearson correlation coefficient was 0.968, whereas, for MRI versus histopathology, it was 0.952. The mean maximum diameter of lesions was slightly larger than that of pathology and the mean maximum diameter of CESM was slightly smaller than that of pathology (both *P* values >0.05). Therefore, CESM can be the better modality for determine the lesion extent than MRI.

Of 77 lesions, CESM detected three cancers that were misdiagnosed as benign lesions by MRI including one intra-cystic papillary carcinoma (2.5 cm) and two DCIS (7 mm and 10 mm). The two DCIS showed characteristics of benign tumors on MRI i.e. regular outline, uniform enhancement, typeIof time density curve and ADC value of 1.28 × 10^−3^ mm^2^/s. The two lesions showed enhancement on combined image and clustered microcalcifications on low-energy image by using CESM. DCIS is breast malignancy that tends to be misdiagnosed by MRI (Teifke et al. 2002; Fallenberg et al. 2014). This may be due to a lack of neoangiogenesis in DCIS, resulting in less enhancement or more unspecific enhancement in MRI. The intra-cystic papillary carcinoma had very few enhanced solid components on MRI, but showed irregular enhanced cyst wall on CESM. Two inflammatory granulomas were misdiagnosed by both CESM and MRI according to routine diagnostic criteria. We reviewed the two cases and speculated that the sign of ring- or honeycomb-like enhancement may provide clues for the diagnosis of inflammatory disease.

There are several limitations in our study. Firstly, the contrast agent for CT application is introduced in CESM. Therefore, CT contrast agent related risks also apply to CESM. Secondly, we didn’t discuss about the radiation dose of CESM. Previous studies have shown that the radiation dose of CESM is about 20 % higher than conventional digital mammography (Dromain et al. 2012; Yaffe and Mainprize 2011). The dose values of CESM meet the recommendations for maximum dose in mammography (Jeukens et al. 2014). We will discuss the risk of breast cancer occurrence by using CESM in further study on large-sample. Thirdly, the enhanced degree was not taken account into the factors of diagnosis. We will discuss the correlation between the enhanced degree and the pathologic outcomes in further research.

In conclusion, CESM is a new diagnostic method for clinics that enables accurate detection and diagnosis of breast lesions. CESM should be a good alternative method of screening breast cancer in high-risk people. However, further studies should be done with a larger number of patients to get more convincing results.
